# Developmental and physiological responses of *Brachypodium distachyon* to fluctuating nitrogen availability

**DOI:** 10.1038/s41598-019-40569-8

**Published:** 2019-03-07

**Authors:** L. C. David, T. Girin, E. Fleurisson, E. Phommabouth, A. Mahfoudhi, S. Citerne, P. Berquin, F. Daniel-Vedele, A. Krapp, S. Ferrario-Méry

**Affiliations:** Institut Jean-Pierre Bourgin, INRA, AgroParisTech, CNRS, Université Paris-Saclay, 78000 Versailles, France

## Abstract

The Nitrogen Use Efficiency (NUE) of grain cereals depends on nitrate (NO_3_^−^) uptake from the soil, translocation to the aerial parts, nitrogen (N) assimilation and remobilization to the grains. *Brachypodium distachyon* has been proposed as a model species to identify the molecular players and mechanisms that affects these processes, for the improvement of temperate C3 cereals. We report on the developmental, physiological and grain-characteristic responses of the Bd21-3 accession of Brachypodium to variations in NO_3_^−^ availability. As previously described in wheat and barley, we show that vegetative growth, shoot/root ratio, tiller formation, spike development, tissue NO_3_^−^ and N contents, grain number per plant, grain yield and grain N content are sensitive to pre- and/or post-anthesis NO_3_^−^ supply. We subsequently described constitutive and NO_3_^−^-inducible components of both High and Low Affinity Transport Systems (HATS and LATS) for root NO_3_^−^ uptake, and *BdNRT2/3* candidate genes potentially involved in the HATS. Taken together, our data validate Brachypodium Bd21-3 as a model to decipher cereal N nutrition. Apparent specificities such as high grain N content, strong post-anthesis NO_3_^−^ uptake and efficient constitutive HATS, further identify Brachypodium as a direct source of knowledge for crop improvement.

## Introduction

The Poaceae family includes cereal crops such as wheat, rice, maize and barley, which provides a major source of food for humans and cattle. It is predicted that by 2050, the human population will increase by 20–50% in parallel to an increase of the living standard and thus, a doubling of grain demand is expected^1–3^. As arable land becomes limited, crop grain yields need to be improved. Nitrogen (N) is a major limiting macronutrient in the fields for crop production and as a consequence, N fertilizers are largely applied in modern agriculture to enhance plant vegetative growth and grain production. From an ecological point of view, the use of large amounts of N fertilizer on fields results in nitrate (NO_3_^−^) leaching, which causes eutrophication and biodiversity depletion. Thus, increasing crop yields while diminishing environmental impacts of agriculture is a major goal of today’s agriculture. However, high-yielding modern cultivars are not suitable, as they have high nutrient requirements, due to their breeding under high nutrient availability^4,5^. Fundamental knowledge on molecular mechanisms of plant nutrition must be increased to engineer new cultivars giving high yields under low nutrient availability.

Mechanisms directly affecting the plant Nitrogen Use Efficiency (NUE) have been largely described at the physiological level in crop species (including wheat, barley, rice and corn) and in the model species, *Arabidopsis thaliana* (Arabidopsis)^6–9^. Most characterizations at the molecular level have however been restricted to the dicot Arabidopsis^10–12^. *Brachypodium distachyon* (Brachypodium) has been proposed as a good model to enhance this knowledge in C3 temperate cereals^13^. This non-domesticated monocot species offers convenient characteristics for academic research (simple diploid sequenced genome, short life cycle, simple growth requirement, large mutant collections, efficient genetic transformation), and is phylogenetically closely related to wheat and barley, enabling an efficient translational approach.

For grain crops, NUE is defined as the ratio between grain yield and available N in the soil per unit of field surface, and is often approximated as the ratio between yield and added N^14^. It is dependent on processes such as N uptake, translocation, assimilation and remobilization^11^. N is mainly taken up from the soil as nitrate (NO_3_^−^) and ammonium (NH_4_^+^) ions, thanks to specialized root transporters. NO_3_^−^ being the most abundant form in aerobic fields, it constitutes the main N source for most temperate crops^11,15–17^. NO_3_^−^ uptake relies on the activity of root High and Low Affinity Transport Systems (HATS and LATS, respectively), involving NO_3_^−^ transporters of the NRT2 and NPF families^18–23^. HATS is active in low nitrate conditions (<1 mM) while LATS is predominant in higher nitrate concentrations. In many species, it has been shown that HATS is partly inducible by nitrate supply subsequently to nitrate deprivation (iHATS). In addition, a constitutive HATS is active under all environmental conditions (cHATS). The induction of HATS by NO_3_^−^ is transient, and long exposure to NO_3_^−^ and/or to other sources of N triggers a repression of NO_3_^−^ influx^24–27^. Furthermore, HATS is a multi-component system since the interaction between NRT2 and its partner protein NRT3 is required for a functional NO_3_^−^ transport activity^28^. In Brachypodium, 5 *BdNRT2* genes have initially been identified by phylogenetic analysis and 2 more have been found in a subsequent analysis^13,29^.

After being taken up from the soil, NH_4_^+^ is mainly assimilated in roots, while NO_3_^−^ is mostly translocated to aerial organs, where it is successively assimilated into nitrite, NH_4_^+^, glutamine and glutamate by the consecutive actions of Nitrate Reductase (NR), Nitrite reductase (NiR) and the GS/GOGAT (Glutamine Synthetase/Glutamate Synthase) cycle^11,30^. Glutamate constitute the precursor of other amino acids and N-containing organic molecules of the plant^11,30^. During plant aging, remobilization processes take place, reallocating N from the source to sink organs^12^. In annual plants, the ultimate remobilization process is the monocarpic senescence (senescence of all vegetative organs), leading to plant death, while grains are filled with reserves^12^. The final NUE can potentially be improved by adjusting these physiological processes.

The potential of enhancing NO_3_^−^ uptake/assimilation or N remobilization for increased grain yield is currently a matter of debate. Studies performed in rice, wheat and maize showed that more than half of the Grain Nitrogen Content (GNC) originate from leaf remobilization, and that this proportion depends on genotypes and soil N availability^31–33^. The importance of remobilization is also highlighted by an increased productivity of stay-green lines, having a delayed senescence^34^. Usually, this yield increase is however associated with a decrease in Grain Protein Content (GPC, directly related to grain N content), which is detrimental to the nutritional and food-processing qualities of the grain. Functional studies of wheat and barley NAM-B1 transcription factors suggest that, in some conditions, a tight control of senescence processes can lead to a higher GPC without decrease in grain yield^35,36^. On the other hand, it has been suggested that enhancing the post-anthesis N uptake is the main way to increase both yield and GPC in wheat^37,38^.

To our knowledge, three studies have been published on N-related mechanisms in Brachypodium. Ingram and colleagues^39^ analyzed the effects of N availability on root architecture and highlighted the variability of the response between accessions. Poiré and colleagues^40^ revealed the stimulation of Brachypodium growth, leaf N content and photosynthetic capacity by high N availability during the vegetative stages. Hong and colleagues^41^ showed that the interaction with symbiotic arbuscular mycorrhiza stimulates the expression of putative NH_4_^+^ transporters, but no effect was seen on N leaf content. As far as we know, no reported study in Brachypodium investigated the dependency of grain yield or GPC on physiological mechanisms such as N uptake, assimilation or remobilization.

Here, we report on a general characterization of Brachypodium physiology and responses to N availability, with a specific focus on grain production. We focussed on the Bd21-3 accession, as it is now the reference accession around the world due to its high transformation efficiency and to the availability of mutants in this background, allowing reverse-genetic approaches. We characterized the effects of NO_3_^−^ availability at both vegetative and reproductive stages on growth, architecture, tissue composition and grain characteristics. We subsequently highlighted the unusual importance of post-anthesis NO_3_^−^ uptake for grain N loading. Finally, NO_3_^−^ transport systems were described at the physiological level, and candidate genes for root high affinity NO_3_^−^ uptake were identified. This data provides a basis for molecular deciphering of NUE-related mechanisms in Brachypodium, as a model for temperate C3 cereals.

## Results and Discussion

### NO_3_^−^ availability at vegetative stage impacts plant growth, development and tissue composition

**Figure 1:**
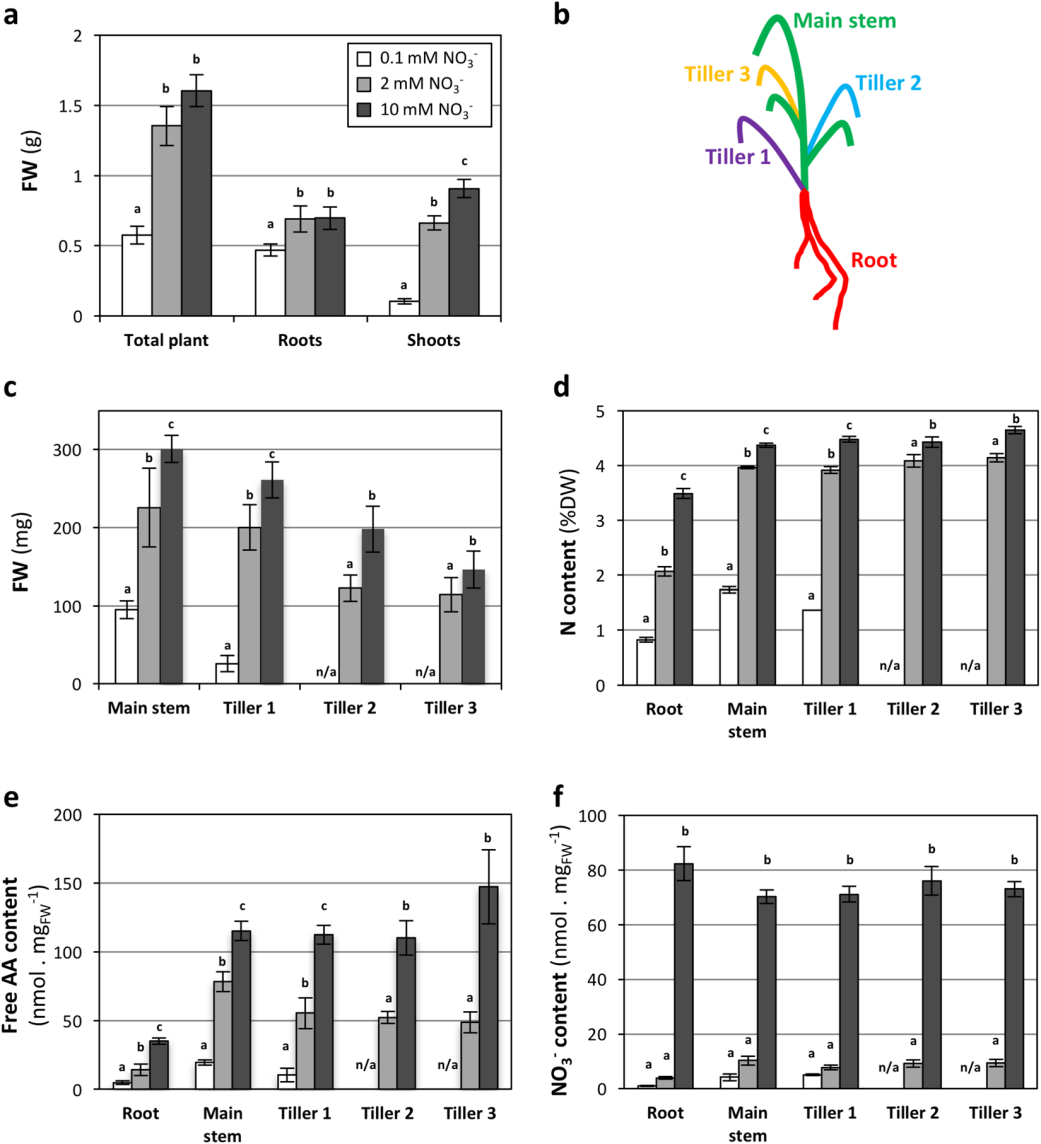
Effects of NO_3_^−^ availability at vegetative stage. Plants were grown on sand for 35 days, watered with nutritive solution containing 0.1, 2 or 10 mM NO_3_^−^. **(a)** Fresh weight of total plant, roots and shoots. **(b)** Schematics of tiller development. Tiller were numbered according to their sequential apparition. **(c)** Fresh weight, **(d)** N content, **(e)** Free AA content and **(f)** NO_3_^−^ content of main stem and tillers. Values correspond to the mean of 4 or 5 biological replicates (2 plants each) +/−SD. Letters indicate statistical groups for each plant part (Non-parametric ANOVA, p < 0.05). N/a: no data available, due to the lack of development of tillers 2 and 3 in the 0.1 mM NO_3_^−^ condition.

The effect of external NO_3_^−^ availability on vegetative growth and development was investigated in 35-days-old plants, grown on sand and watered with a nutritive solution containing 0.1, 2 or 10 mM NO_3_^−^ as sole source of N. The total fresh weight of plants grown on 0.1 mM NO_3_^−^ was reduced when compared to the other conditions (64% reduction as compared to 10 mM NO_3_^−^). No significant difference was observed between plants grown on 2 and 10 mM NO_3_^−^ (Fig. 1a; Supplementary Information Fig. S1). A similar effect was observed on the root fresh weight. In contrast, a reduction of shoot fresh weight (Fig. 1a) was already observed on 2 mM NO_3_^−^ as compared to 10 mM NO_3_^−^, and more severe on 0.1 mM NO_3_^−^. Brachypodium Bd21-3 shoot biomass was thus more sensitive to NO_3_^−^ limitation than root biomass, corresponding to the well documented decrease of shoot/root ratio under N limiting conditions (Supplementary Information Fig. S1b) in a variety of plants^42,43^, and to a previous study on Brachypodium Bd21-3^40^.

Tillering is a primordial characteristic of cereals, as it influences the number of spikes and thus the grain yield. To further characterize the effect of N availability, the main stem and the different tillers (Fig. 1b) were separated. At 0.1 mM NO_3_^−^, only 40% of the plants developed a first tiller (data not shown) and none developed tillers of higher ranks. In the other conditions, all plants produced 3 tillers, in addition to the main stem. This effect of N availability on tiller number is consistent with observations made in wheat and barley^44–46^. As for total shoot, the fresh weight of the main stem and of tillers of different ranks were progressively reduced with the reduction of NO_3_^−^ availability (Fig. 1c). Thus, both shoot growth and development were limited by low N availability on 0.1 mM NO_3_^−^, whereas only shoot growth was affected on 2 mM NO_3_^−^, with a similar effect on the main stem and tillers.

To characterize the effect of NO_3_^−^ availability on the plant, Nitrogen (N), Carbon (C), free amino acids (AA) and NO_3_^−^ contents of the tissues were measured. Nitrogen content was reduced in all parts of the plant (roots, main stem and tillers) on both 0.1 and 2 mM conditions as compared to 10 mM NO_3_^−^, with a stronger effect on 0.1 mM (Fig. 1d). The nitrate availability had a minor effect on C content (Supplementary Information Fig. S1c). Consequently, C/N ratio was reduced by increasing NO_3_^−^ availability, with similar patterns for the different plant parts (Supplementary Information Fig. S1d). The effect on AA content was qualitatively similar to that observed on N content, with significant differences between the three conditions, and comparable effects on all parts of the plant (Fig. 1e). Free AA content was quantitatively more affected than N content at the whole plant level, with 91% and 55% global decrease on 0.1 mM and 2 mM, respectively, when compared to 10 mM (whole plant N content was reduced in parallel by 76% and 25%) (Supplementary Information Fig. S1e,f). Nitrate content was also strongly affected in all plant parts by low external NO_3_^−^ availability (Fig. 1f), being globally reduced by 98% and 91% on 0.1 and 2 mM, respectively (Supplementary Information Fig. S1g).

Thus, external NO_3_^−^ limitation led to a strong decrease of internal NO_3_^−^ pools and a strong to moderate decrease of both free AA and N contents, leading to a moderate effect on plant growth and development. Interestingly, the main stem and initiated tillers responded similarly to reduced NO_3_^−^ availability, suggesting there is no prioritization for N allocation between main stem and tillers.

### Both pre- and post-anthesis NO_3_^−^ availability affect vegetative growth and grain yield

Based on the effects seen at the vegetative stage, we further investigated the effects of pre- and post-anthesis N availability on end of cycle plant and grain characteristics. Plants were grown hydroponically on a medium containing NO_3_^−^ as a sole source of N. Control plants (Ctrl) were maintained on media containing 1 mM NO_3_^−^. This condition was chosen based on the previous experiment. This nitrate concentration should lead to intermediate growth and N physiological state of the plants, enabling to test both positive and negative effects of changes in external NO_3_^−^ availability. Four treatment conditions were used: plants grown on media containing either low N (LN; 0.1 mM NO_3_^−^) or high N (HN; 10 mM NO_3_^−^) until anthesis of the first spikes, then transferred to 1 mM NO_3_^−^; and conversely, plants grown on 1 mM NO_3_^−^ until anthesis, then transferred to LN or HN. Plants grown in the three different conditions at vegetative stage sequentially reached the anthesis stage (Supplementary Information Fig. S2a). Plants grown at vegetative stages on 0.1 and 1 mM NO_3_^−^ exhibited similar fresh weights at anthesis, despite a shift to reach this stage, whereas plants grown on 10 mM NO_3_^−^ were more developed (Supplementary Information Fig. S2b).

**Figure 2:**
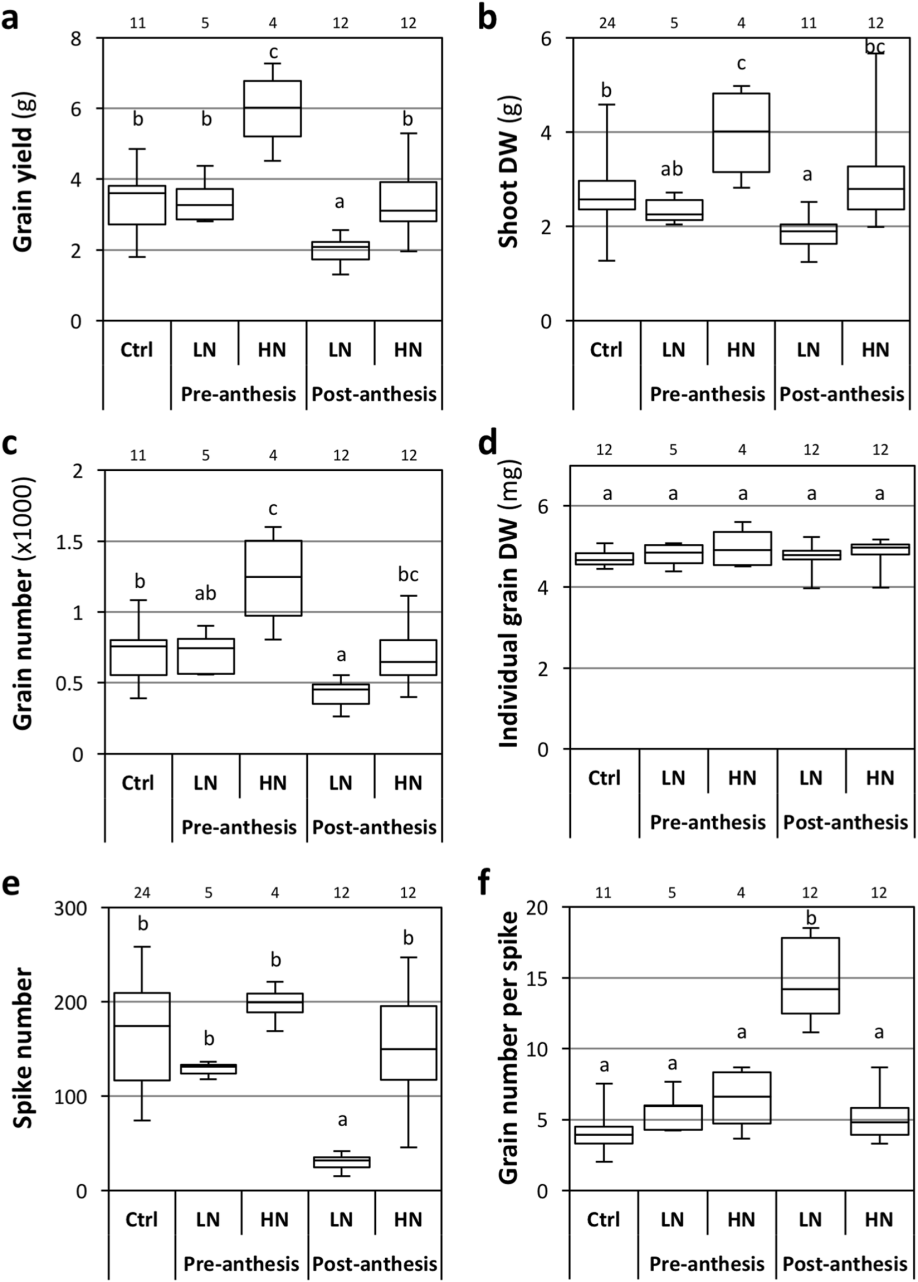
Effects of pre- and post-anthesis NO_3_^−^ availability on end of cycle characteristics. Plants were grown in hydroponics. Ctrl: control plants grown on 1 mM NO_3_^−^ during the entire life cycle. Pre-anthesis: plant grown on 0.1 or 10 mM NO_3_^−^ (LN and HN, respectively) until anthesis, and subsequently on 1 mM NO_3_^−^. Post-anthesis: plant grown on 1 mM NO_3_^−^ until anthesis, and subsequently on LN or HN. **(a)** Grain yield per plant. **(b)** Shoot Dry Weight (DW). **(c)** Grain number per plant. **(d)** DW of individual grain. **(e)** Spike number per plant. **(f)** Grain number per spike. The boxplots represent minimum, 1^st^ quartile, median, 3^rd^ quartile and maximum values. Letters indicate statistical groups (Non-parametric ANOVA, p < 0.05). Number of biological replicates for each condition are indicated above the charts.

At the end of the cycle, grain yield per plant was increased by pre-anthesis HN treatment (+68%), reduced by post-anthesis LN treatment (−42%), and not affected in other conditions (Fig. 2a). The shoot dry weight followed a similar pattern (+56%, −26% and no effect, respectively; Fig. 2b). Accordingly, the harvest index (grain/total above-ground dry weights) was not affected by the treatments as compared to the control condition (Supplementary Information Fig. S2c), suggesting that the variations in grain yield are a consequence of variations in vegetative growth. In barley and wheat, grain yield is similarly dependent on the vegetative biomass, which related to the nitrogen fertilization^44,47^. In contrast, the effect of post-anthesis N availability (LN treatment) on grain yield and shoot biomass was surprising, as it is usually not observed in cereals^37,48,49^. This was linked to the continuation of shoot growth after anthesis, whereas it is generally admitted that vegetative growth stops around anthesis stage in cereals^37^. The maintenance of vegetative growth after anthesis in Brachypodium Bd21-3 might be linked to the undomesticated nature of the species. In addition, it could have been enhanced by the growing conditions used in our study, as it has been reported that tillering hydroponic-grown wheat can continue for an indefinite time, under non-limiting conditions^44^.

Plants grown on 10 mM NO_3_^−^ up to anthesis and subsequently on 1 mM NO_3_^−^ presented higher end-of-cycle shoot biomass and grain yield than the control plants. In contrast, shoot biomass and grain yield were not stimulated by the 10 mM post-anthesis treatment when compared to the control, and were reduced by the 0.1 mM post-anthesis treatment. Thus, in our conditions, 1 mM NO_3_^−^ was sufficient for maximal growth in late, but not in early development. This is consistent with the observation made by Gastal and Lemaire^50^, that the N uptake demand from the plant to achieve maximal growth decreases as the plant gets bigger. Our observation might thus reflect the negative relationship between plant biomass and N supply necessary for maximum growth, known as the critical N curve and observed for many crops^50,51^.

### Grain yield depends on grain number and grain abortion rate

Grain yield in C3 temperate cereals is known to be affected by environmental conditions through both grain number and grain size^52–55^. Thus, we explored the effects of N availability on these traits. The grain number per plant was increased by pre-anthesis HN treatment and reduced by post-anthesis LN treatment (+65% and −40%, respectively; Fig. 2c), whereas the individual grain weight was not affected (Fig. 2d). This established a direct link between grain yield and grain number. Such an absence of an effect of N nutrition on grain weight has previously been described for wheat grown in pots or in hydroponics^44,56^. It is thus possible that grain weight is more sensitive to N availability in the field than in controlled conditions.

We subsequently analyzed the basis of the variation of grain number. The pre-anthesis HN treatment did not statistically increase the number of spikes (equal to the number of tillers, as all tillers developed spikes in the 5 conditions), nor the grain number per spike (Fig. 2e,f). This suggests that the increased grain yield in this condition is due to slight multiplicative increases in both the number of tillers and the grain number per spike. On the other hand, the yield decrease in the post-anthesis LN treatment correlated with a strong reduction of spike number (−82%; Fig. 2e) and a high increase in average grain number per spike (+261%; Fig. 2f). This increase in grain number per spike was due to a reduction of grain abortion to 25% of grain initiation, as compared to 53% in the control condition (Supplementary Information Fig. S2d). Thus, the post-anthesis initiation of tillers was restricted by low N availability, but grain production was partially compensated by a greater development of the spikes. Interestingly, the general high proportion of grain abortion confirms the observation by Oscarson^44^, stating that in most N conditions, wheat plants produce slightly more tillers than can be supported for grain filling. The author hypothesized that producing extra tillers enables the plant to produce more grains in case of a late increase in nutrient availability.

### Post-anthesis N availability affects N and protein contents and free AA composition of the grains

**Figure 3:**
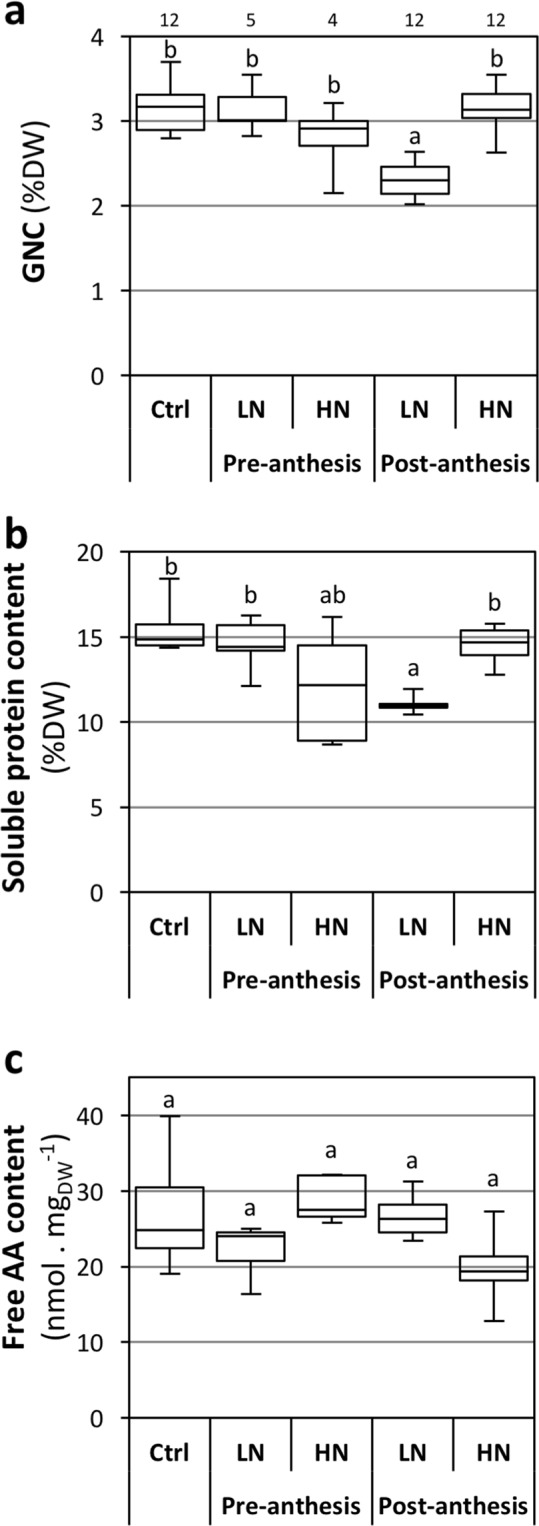
Effects of pre- and post-anthesis NO_3_^−^ availability on grain composition. Growing conditions and treatments were as described in Fig. 2. LN, Ctrl and HN: 0.1, 1 and 10 mM NO_3_^−^, respectively. Grain contents in **(a)** total N (GNC), **(b)** extractable proteins and **(c)** free AA. The boxplots represent minimum, 1^st^ quartile, median, 3^rd^ quartile and maximum values. Letters indicate statistical groups (Non-parametric ANOVA, p < 0.05). Number of biological replicates for N content are indicated above the chart; other charts are based on 5 biological replicates per condition.

We investigated the effect of the pre- and post-anthesis N treatments on grain composition, including N and protein contents. Grain protein content (GPC), directly related to grain N content (GNC), is a main criterion for cereal grain quality, in relation to the end-use. For malting barley, the GPC should be in the range of 8.5–12.5% DW (corresponding to GNC of 1.4–2% DW), and can be higher for feed barley^57,58^. A high protein content is favorable for the baking quality of wheat flour^59^, and GPC between 10–15% are thus usually reported for bread wheat grains (corresponding to GNC of 1.75–2.6%)^8,37,60,61^. Recently, high GNC (ranging 2.3–3.0%) has been reported in bread wheat grown in a semi-hydroponic system^49^. In our experiment, GNC was around 3% DW (Fig. 3a), corresponding to an estimated GPC of 17% DW when using the accepted conversion factor of 5.7^62^. Our results are in agreement with the high GNC (2.9–3.7%) reported for Brachypodium Bd21-3 in different growth conditions^63,64^. These results underline the generally high grain N content of Brachypodium Bd21-3, and identify this species (or at least this accession) as a source of knowledge for the improvement of bread wheat GPC.

The GNC was quite unaltered by pre- and post-anthesis N treatments (Fig. 3a), as it was statistically affected only by the post-anthesis LN treatment (−27%). In combination with the reduction of grain yield in this condition, this highlights the sensitivity of Bd21-3 to low N availability for grain quality and quantity during the reproductive phase. Interestingly, GNC was unaffected by the pre-anthesis HN treatment that triggered an enhanced grain yield, underlining independency between the traits. Cereal GNC is known to be highly dependent on post-anthesis N uptake and on remobilization from vegetative organs of N assimilated before anthesis^37,48,65^. Accordingly, a late field application of fertilizer is usually recommended to improve grain N uptake during grain filling, and thus grain protein content^66^, as observed in our experiment. The source limitation of GNC was apparent for low N treatment, showing that N remobilization could not compensate the reduction of external N availability. This is in agreement with the dominance of post-anthesis uptake over remobilization for grain N filling in our conditions (see thereafter).

Compared to the control condition, the grain C content was marginally reduced under all treatments, except by the pre-anthesis LN treatment (Supplementary Information Fig. S2e). The C/N ratio was only significantly modified in the post-anthesis LN treatment (Supplementary Information Fig. S2f). The grain content in soluble proteins followed the same pattern as the GNC, with only the post-anthesis LN condition presenting a significant difference with the control (−26%; Fig. 3b). The grain soluble protein content ranged between 10–15%, which is consistent with published studies in Brachypodium Bd21-3^63,64^. The grain composition in soluble protein was visualized by SDS PAGE (Supplementary information Fig. S3). The protein profile was consistent with a previous report on Brachypodium grain protein composition^63^, and no major differences in the protein profiles was found between the treatments.

**Figure 4:**
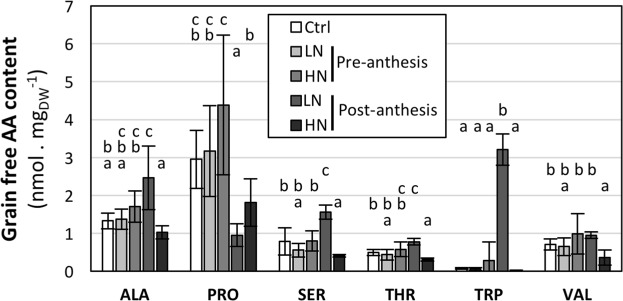
Effects of pre- and post-anthesis NO_3_^−^ availability on grain free AA composition. Growing conditions and treatments were as described in Fig. 2. LN, Ctrl and HN: 0.1, 1 and 10 mM NO_3_^−^, respectively. Only free AA levels affected by the treatments are presented; see Table S1 (Supplementary Information) for full set of data. Values are means of 5 biological replicates +/−SD. Letters indicate statistical groups for each AA (Non-parametric ANOVA, p < 0.05).

Although grain free amino acid (AA) content represents a small proportion of total grain N content (less than 1%)^67^ when compared to seed protein (around 60%), it could indicate an effect of N availability on grain N storage capacity^68^. The free AA pool results from the balance between neo-synthesis in grain and AA remobilization from leaves on the one hand, and seed protein synthesis during grain development on the other. In our experiment, no significant change in total free AA content was observed (Fig. 3c), suggesting an absence of global nitrogen imbalance between grain AA supply and seed protein synthesis. In contrast, the analysis of the free AA composition revealed some specific changes. For instance, Asn (27–44% of free AA), Pro (4–17%), Gln (5–10%), Ala (5–10%), Asp (5–7%), Ser (3–6%), Glu (3–6%) and Arg (3–6%) represented the main free AA’s in the grain (Supplementary Information Table S1). Post-anthesis treatments induced significant changes in the content of some of the free AA’s (Fig. 4). Post-anthesis LN treatment triggered a large increase in Trp (x45), moderate increases in Thr, Ser, and Ala, and a decrease in Pro (x1/3) (Fig. 4). Post-anthesis HN treatment triggered moderate decreases (x1/2) in Thr, Ser and Val. The effects of post-anthesis treatments on the free AA pool may result from specific variations in N remobilization or protein synthesis.

The storage proteins in Brachypodium Bd21-3 grains are composed mainly of globulins (50–60%) and prolamins (12%), similar to that in rice and oat^64^. The major AA’s of grain proteins are Glx, Asx, Pro and Gly (20%, 10%, 8.6% and 8.5% respectively)^64^. In wheat, accumulation of prolamins correlates with the post-anthesis N supply^65,69^. Interestingly, Brachypodium prolamins are homologous to wheat avenin-like and γ-gliadins, the latter being characterized by a high content in Pro (17.4%)^64^. Thus, the decrease of grain free Pro content in our post-anthesis LN treatment could correlate with a limitation in prolamin synthesis. Additionally, the lower protein level observed in post-anthesis LN suggests that N fertilization has the same effect on prolamin as it does in wheat. The other major free AAs (Glx, Asx, and Gly) found in grain proteins of Brachypodium Bd21-3 did not show significant changes in the free AA pool. Some less abundant amino acids (Thr, Ser, Ala, and Val) were increased by post-anthesis N limitation and/or decreased by post-anthesis N abundance, thus were likely not limiting for globulin synthesis. Trp is an essential amino acid for human and animal nutrition and is usually in limiting concentrations in wheat grain. We observed a spectacular increase of Trp in the grains after post-anthesis LN treatment. Trp was below the detection threshold in a previous study^64^, due to the methodology (acidic hydrolysis of protein) that degrades this AA. Trp content in prolamin varies between grain cereals, being lower in wheat prolamin than in the globulin fraction^67,70^. Thus, our results highlight a limitation in seed protein synthesis, probably in prolamin but also in globulin after post-anthesis LN treatment.

### Grain N loading depends primarily on post-anthesis N uptake

Our data highlighted an important correlation of both grain quality and quantity with post-anthesis NO_3_^−^ availability (Figs 2 and 3), thus we further characterized N fluxes in the plant at this stage. N grain loading in cereals is dependent on both N remobilization from vegetative organs (taken up before anthesis) and post-anthesis N uptake^37,48,65^. We thus quantified both processes and investigated their coordinated responses to variations in NO_3_^−^ availability. Plants were grown hydroponically on media containing 0.2 mM NO_3_^−^ until anthesis of the first spikes, then transferred to conditions of either N deprivation, limitation or abundance (0.01 (A), 0.2 (B) and 10 (C) mM NO_3_^−^, respectively). These contrasting conditions were chosen to maximize the effect of the treatments on both grain yield per plant and GNC (Supplementary Information Fig. S4). A pulse-chase strategy was used at the vegetative phase (replacement of the NO_3_^−^ by ^15^NO_3_^−^ for 5 days) to enrich the vegetative tissues with ^15^N stable isotope. Plants were harvested either at anthesis (to map the ^15^N distribution at the end of vegetative phase) or after full senescence of the plant. Roots, shoots and spikes were collected separately, and dry weight, N content and ^15^N enrichment were measured (Supplementary Information Table S2). Nitrogen fluxes between plant parts after anthesis were then calculated as previously described^71^.

**Figure 5:**
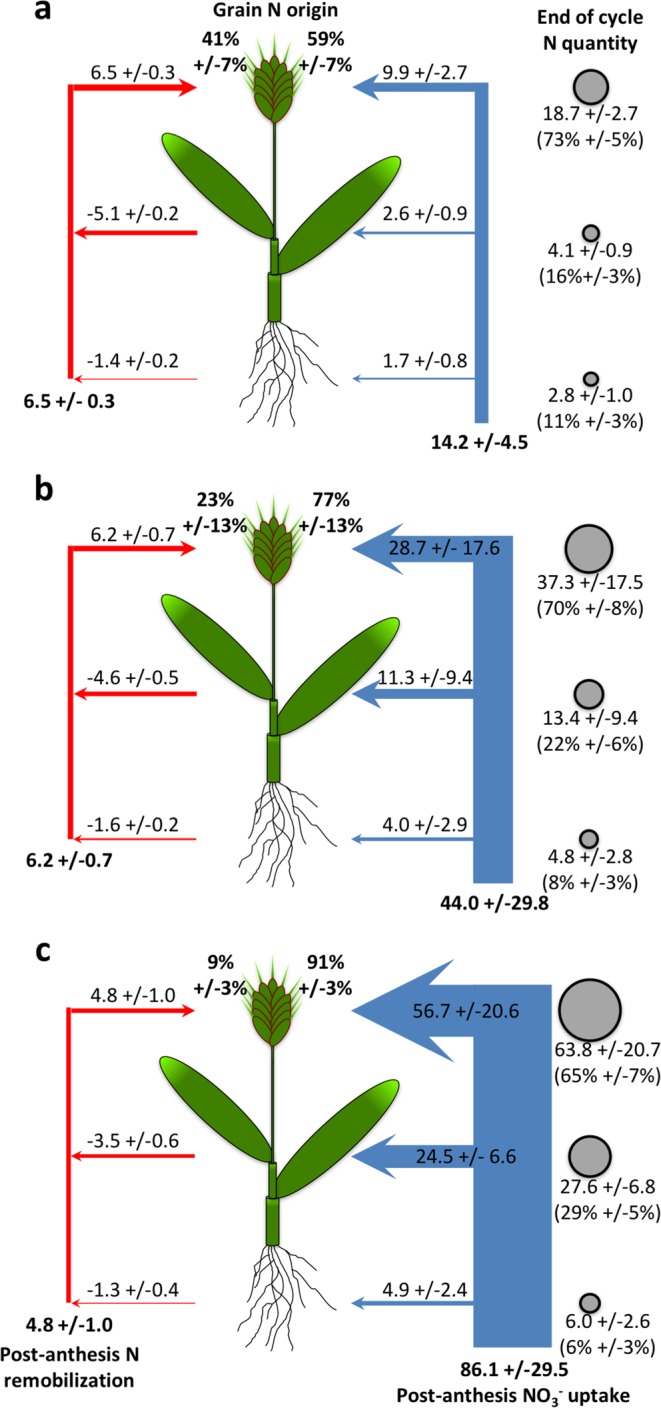
Effect of post-anthesis NO_3_^−^ availability on N fluxes. Plants were grown hydroponically on 0.2 mM NO_3_^−^ and transferred at anthesis to **(a)** 0.01, **(b)** 0.2 or **(c)** 10 mM NO_3_^−^. Left arrows (red): post-anthesis remobilization of pre-anthesis N pools from vegetative plant parts. Right arrows (blue): post-anthesis NO_3_^−^ uptake. Arrows indicate the N fluxes from/to spikes, shoots or roots; values of the fluxes (mg_N_) are specified above the arrows. Bold values at the bottom of the panels correspond to total N remobilization (left) and total NO_3_^−^ uptake (right) at the plant level (mg_N_). Percentages above the spikes indicate the proportion of grain N originating from remobilization (left) and from post-anthesis uptake (right). Total N quantities in spikes, shoots and roots after full senescence of the plants are indicated by circles on the right of each panel; absolute values (mg_N_) are indicated; distribution of N quantity between spikes, shoots and roots are specified in brackets (% of the plant total N). Values correspond to the mean of 5 (panel a) or 7 (panels b and c) biological replicates +/−SD. Raw data (DW, N content and ^15^N enrichment of the different plant parts) and results of statistical tests are presented in Supplementary tables ST2 and ST3, respectively.

The post-anthesis NO_3_^−^ uptake was stronger than remobilization fluxes from vegetative shoots and roots in all conditions, and was highly stimulated by NO_3_^−^ availability (Fig. 5; see Supplementary Information Table S3 for statistical tests). Hence, end of cycle grain N originated mainly from NO_3_^−^ taken up at the post-anthesis stage (59% to 91%, in deprivation and abundance conditions, respectively). Grain N originating from remobilization was significantly reduced under high N availability, both on relative values (9% and 41% of grain N under abundance and deprivation, respectively) and on absolute quantities (4.8 and 6.5 mg_N_), reflecting a negative effect of N availability on N remobilization. The proportion of total plant N localized in the grain at the end of the cycle was relatively high in all conditions, ranging from 65% under abundance to 73% under deprivation (Fig. 5). This was consistent with the strong allocation of post-anthesis N uptake to the grain (65% to 70%).

In cereals such as wheat, barley, rice and maize, remobilization is regarded as the main N source for grain loading, providing 60–90% of the final grain N, depending on genotypes and environmental conditions^32,33,48,56,72^. In our experiment, remobilization had a minor role, furnishing 9–41% of grain N depending on post-anthesis NO_3_^−^ availability. A low importance of remobilization has recently been described in wheat: out of 8 measurements (2 genotypes grown in 4 conditions, including low and high N availability), 7 ranged from 0% to 37%, and only one had a value above 50%^49^. This study, based on plants grown in a semi-hydroponic system in a growth chamber, could suggest that our observed low values are linked to our experimental setup. Alternatively, it has been reported that a low pre-anthesis and/or a high post-anthesis N availability reduce the contribution of remobilization for grain loading^49,73,74^, which may be the case in our study. Finally, it is also possible that grain N loading in Brachypodium Bd21-3 is mainly based on post-anthesis N uptake, rather than on N remobilization. A similar case has been reported for instance in sorghum^75,76^ and in stay-green maize genotypes^77^. In the case of Brachypodium, the relative importance of post-anthesis NO_3_^−^ uptake could be related to the non-domesticated nature of the species. Investigating several Brachypodium accessions, each grown in various conditions, would be necessary to confirm whether a genetic and/or environmental effect is the reason for this high contribution of post-anthesis NO_3_^−^ uptake for grain N loading.

High external NO_3_^−^ availability had a strong positive effect on NO_3_^−^ uptake and a weak, but significant, repressive effect on N remobilization from shoots to grains (6.2 mg_N_ on 0.2 mM NO_3_^−^ *versus* 4.8 mg_N_ on 10 mM NO_3_^−^). This reduced remobilization in situations of high post-anthesis N uptake have frequently been observed in wheat^37,49,78–80^. As highlighted by Bancal^81^, the difference in remobilization has a limited impact on the N distribution to the grain (1.4 mg_N_ difference in remobilization between 0.2 and 10 mM conditions, to be compared to a total grain N content of 37.3 and 63.8 mg_N_; Fig. 5). Accordingly, the NO_3_^−^ availability had no statistically significant effect on N partitioning (73% of the total plant N was located in the grain on 0.01 mM NO_3_^−^, and 65% on 10 mM NO_3_^−^). The Nitrogen Harvest Index (NHI; proportion of above-ground N localized in the grains) was between 70% and 82% (on 10 mM and 0.01 mM NO_3_^−^, respectively), which is comparable to values observed in wheat, barley and oilseed rape^8,61,82^, and much higher than in Arabidopsis (25–53%)^7^.

### NO_3_^−^ uptake is mediated by constitutive and inducible HATS and LATS

Due to the importance of NO_3_^−^ uptake for grain N loading, we further investigated characteristics of NO_3_^−^ influx. In most studied plants – such as barley, wheat, Arabidopsis and *Brassica napus* – root NO_3_^−^ uptake is performed by three classes of systems: constitutive High Affinity Transport Systems (cHATS), inducible HATS (iHATS) and constitutive Low Affinity Transport System (cLATS)^26,83–87^.

**Figure 6:**
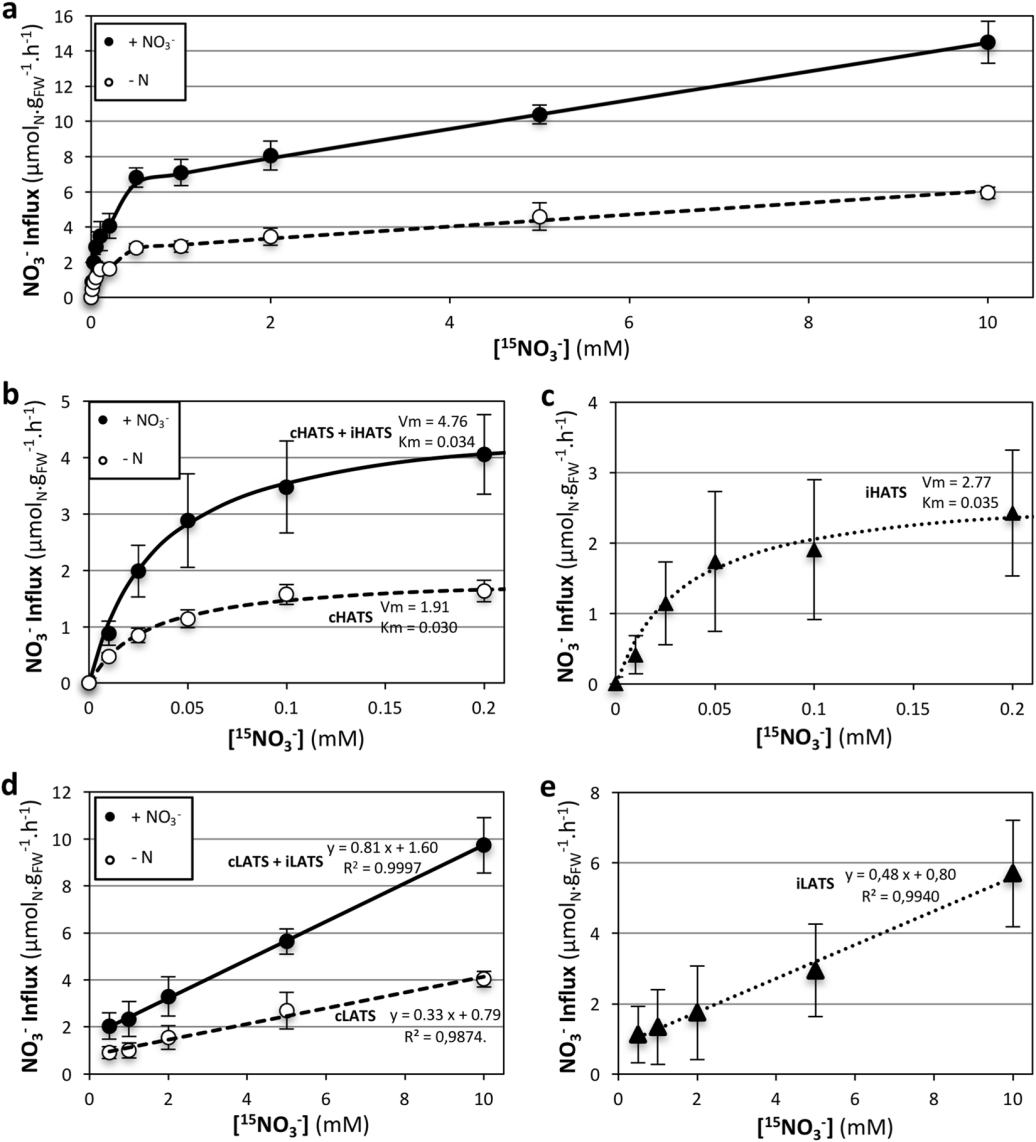
Characterization of High and Low NO_3_^−^ Transport Systems (HATS and LATS, respectively). Plants were grown hydroponically on 2 mM NO_3_^−^ for 7 days, then N starved for 8 days (−N, open circles and dashed lines) or N starved for 6 days and induced with 1 mM NO_3_^−^ for 24 h (+NO_3_^−^, closed circles and solid lines). **(a)** ^15^NO_3_^−^ influx at external concentrations ranging from 0.01 to 10 mM ^15^NO_3_^−^. **(b)** ^15^NO_3_^−^ influx at external concentrations ranging from 0.01 to 0.2 mM ^15^NO_3_^−^. HATS kinetics were fitted with Michaelis-Menten curves (*Influx* = *Vmax* * [^15^*NO*_3_^−^]/(*Km* + [^15^*NO*_3_^−^]). Vmax and Km values were obtained by linear regression on the Lineweaver-Burk representation (*1/Influx* = *a* * (*1*/[^15^*NO*_3_^−^]) + *b*, with *Vmax* = *1*/*b* and *Km* = *a/b*). cHATS and iHATS: constitutive and inducible HATS (respectively). **(c)** iHATS influx at external concentrations of 0.01–0.2 mM ^15^NO_3_^−^, obtained by subtracting −N values from +NO_3_^−^ values in panel b and fitted with Michaelis-Menten curve. **(d)** LATS ^15^NO_3_^−^ influx at external concentrations ranging from 0.5 to 10 mM ^15^NO_3_^−^, obtained by subtracting HATS Vmax values (panel b) from corresponding influx values (panel a). LATS kinetics were fitted with linear curves. cLATS and iLATS: constitutive and inducible LATS (respectively). (**e**) iLATS curve, obtained by subtracting values from −N condition to values from +NO_3_^−^ condition in panel d, fitted with a linear curve. Values correspond to the mean of 5 biological replicates +/−SD.

N-deprived plants grown hydroponically were either induced with 1 mM NO_3_^−^ for 24 h or remained uninduced. Root NO_3_^−^ influx was then measured at external concentrations ranging from 0 to 10 mM. Influx was enhanced by the NO_3_^−^ pre-treatment for all the tested concentrations, highlighting the inducibility of the uptake capacity (Fig. 6a). The influx curves were composed of two phases, indicating the involvement of HATS and LATS. HATS was active at low NO_3_^−^ concentration, reaching saturation at around 0.2 mM NO_3_^−^ (Fig. 6b). Constitutive HATS (cHATS) was active in the absence of NO_3_^−^ pre-treatment, whereas both cHATS and inducible HATS (iHATS) contributed to the activity of HATS after NO_3_^−^ pre-treatment (Fig. 6b). Both cHATS and iHATS followed Michaelis-Menten kinetics, with similar Vmax and Km values (Fig. 6b,c). The induction by NO_3_^−^ of HATS was relatively low (factor 2.5) compared to other studies, where induction factors of 5–30 are often reported^26^. Interestingly, cHATS Vmax was higher than what has been described in wheat and barley in similar conditions (1.9 compared to 0.3–0.8 µmol_N_.g_FW_^−1^.h^−1^), whereas cHATS + iHATS Vmax corresponded to the low range seen in wheat and barley (4.8 compared to 4–12 µmol_N_.g_FW_^−1^.h^−1^)^26,83,88–90^. This suggests that the HATS is characterized by both a strong basal activity and a relatively weak inducibility by NO_3_^−^ in Brachypodium Bd21-3. These characteristics might reveal an adaptation to N-poor natural habitats, in accordance with the origin of this accession from an arid region in Iraq, a typical ecosystem where N availability is low^91,92^.

LATS activity was calculated by subtracting the Vmax values of the HATS from the corresponding measured influx in the range 0.5–10 mM NO_3_^−^. Surprisingly, the LATS activity was induced by NO_3_^−^ pre-treatment (Fig. 6d). Both constitutive LATS (cLATS) and inducible LATS (iLATS) followed a linear curve in the studied range (Fig. 6d,e). This existence of iLATS has not been observed in most species including barley, in which extensive studies have been performed^26,83,84,93^. To our knowledge, an iLATS has only been unambiguously reported in wheat and in *Populus tremuloides*^94,95^. This highlights the suitability of Brachypodium Bd21-3 as a model for NO_3_^−^ uptake systems in wheat.

### HATS is regulated by N availability, likely involving *BdNRT2/3* genes

**Figure 7:**
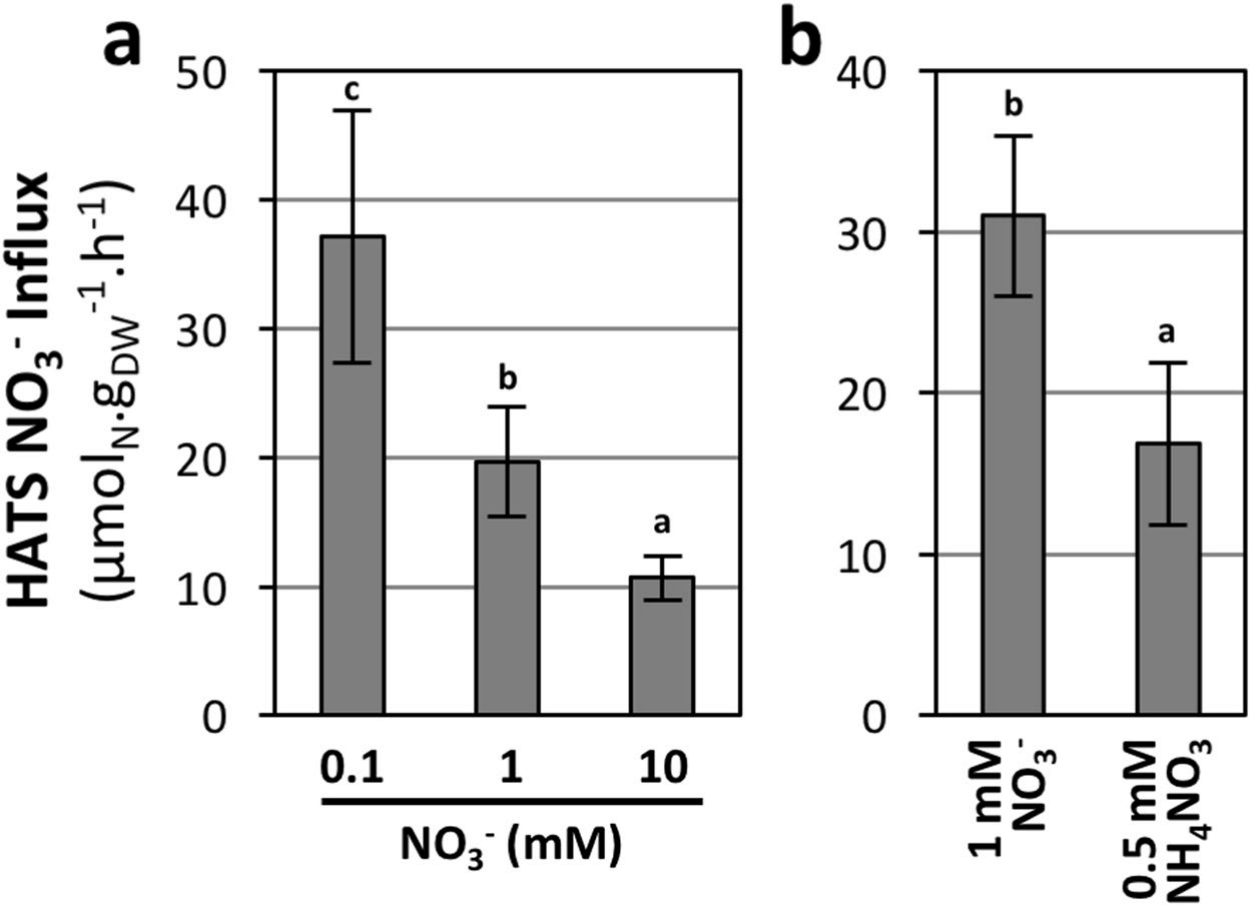
Responses of HATS to N availability. HATS was quantified at 0.2 mM ^15^NO_3_^−^ on 17-day-old plants grown hydroponically on either **(a)** 0.1, 1 or 10 mM NO_3_^−^ or **(b)** 1 mM NO_3_^−^ and transferred for 24 h to 1 mM NO_3_^−^ or 0.5 mM NH_4_NO_3_. Values correspond to the mean of 5 biological replicates +/−SD. Letters indicate statistical groups (Non-parametric ANOVA, p < 0.05).

High affinity NO_3_^−^ transport systems are of prime importance for crop improvement, as ideal varieties should have a high uptake capacity at low NO_3_^−^ availability. The regulations of HATS activity were further investigated by cultivating plants under contrasting conditions of steady state N availability or by treating them for 24 h with NH_4_NO_3_. HATS activity was then measured by transferring the plants for 5 minutes on 0.2 mM ^15^NO_3_^−^ nutrient solution. HATS influx was gradually reduced by increasing NO_3_^−^ availability from 0.1 to 10 mM NO_3_^−^ (Fig. 7a). Similarly, HATS activity was repressed by the presence of ammonium (NH_4_^+^) in the medium (Fig. 7b). This is consistent with the repression of HATS by increased levels of NO_3_^−^ or by other sources of N, described in other species^24–27^.

**Figure 8:**
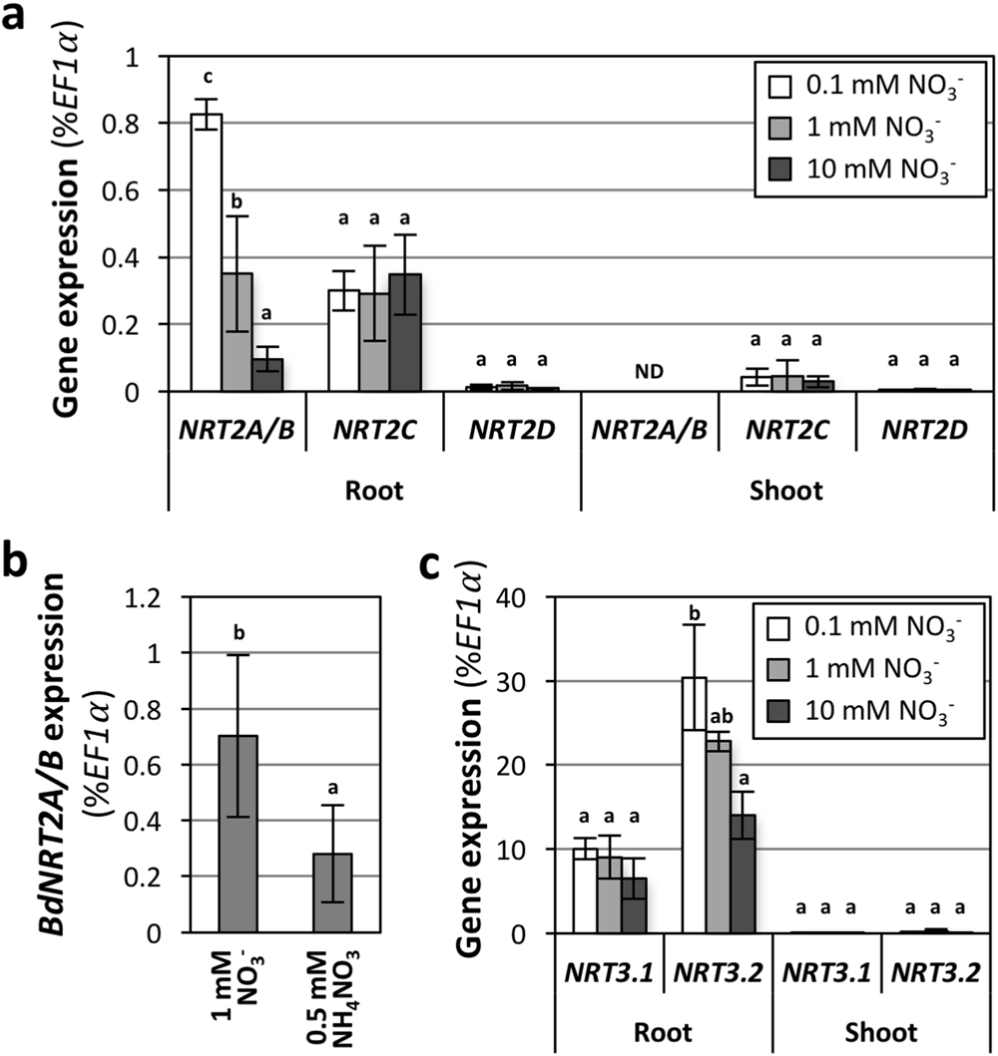
Response of *BdNRT2/3* expression to N availability at vegetative stage. Plants were grown in hydroponics for 17 days under the indicated N condition. **(a)** Response of *BdNRT2* genes to NO_3_^−^ availability. *BdNRT2A* and *BdNRT2B* were quantified simultaneously due to high sequence homology. ND: not detected. **(b)** Response of *BdNRT2A/B* expression to NH_4_^+^. **(c)** Response of *BdNRT3* genes to NO_3_^−^ availability. Values are normalized by *BdEF1α* expression; similar results were obtained after normalization by *BdUBC18* or *BdUbi10*. Values correspond to the mean of 4–5 biological replicates +/−SD. Letters indicate statistical groups for each gene and plant organ system (ANOVA on Log2-transformed values, p < 0.05).

HATS has been described in several species as mediated by NRT2 transporters, in interaction with NRT3^28,96^. We previously identified seven *NRT2* and two *NRT3* genes in Brachypodium^13^. We investigated the expression of four co-orthologs (*BdNRT2A* to *BdNRT2D*) of the major *NRT2* genes identified in Arabidopsis and barley (*AtNRT2*.*1* and *HvNRT2*.*1*, respectively). Due to nearly identical coding sequences of *BdNRT2A* and *BdNRT2B* in Bd21-3 accession, these genes were quantified simultaneously. The quantification of gene expression was done by RT-qPCR in parallel to the characterization of HATS regulations (above), on independent plants. The investigated genes were more expressed in roots than in shoots (Fig. 8a). Whereas *BdNRT2C* and *BdNRT2D* expressions were insensitive to NO_3_^−^ availability, *BdNRT2A/B* were repressed by high NO_3_^−^ (Fig. 8a) and NH_4_^+^ (Fig. 8b), as observed for the HATS (Fig. 7). *BdNRT3* genes were also mainly expressed in the roots (Fig. 8c). Expression of *BdNRT3*.*1* was unchanged under all NO_3_^−^ supplies, whereas *BdNRT3*.*2* was repressed by high NO_3_^−^ concentrations (Fig. 8c). *BdNRT2A/B* and *BdNRT3*.*2* thus constitute good candidates to be involved in HATS activity in these conditions in Brachypodium Bd21-3, as they are mainly expressed in the roots, and their expression pattern follows the HATS activity.

**Figure 9:**
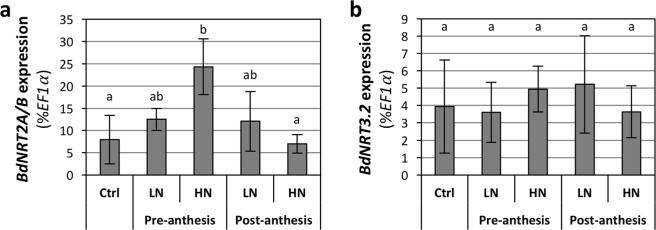
Effects of pre- and post-anthesis NO_3_^−^ availability on root *BdNRT2/3* expression at reproductive stage. Growing conditions and treatments were as described in Fig. 2. LN, Ctrl and HN: 0.1, 1 and 10 mM NO_3_^−^, respectively. Plants were harvested 2 weeks after anthesis. **(a)** *BdNRT2A/B* expression. The genes were quantified simultaneously due to high sequence homology. **(b)** *BdNRT3*.*2* expression. Values are normalized by *BdEF1α* expression; similar results were obtained after normalization by *BdUBC18* or *BdUbi10*. Values correspond to the mean of 4–5 biological replicates +/−SD. Letters indicate statistical groups (ANOVA on Log2-transformed values, p < 0.05).

The expression of *BdNRT2A/B* and *BdNRT3*.*2* was also investigated at post-anthesis stage in the experiment dedicated to the characterization of the effects of NO_3_^−^ availability pre-anthesis and post-anthesis. *BdNRT2A/B* expression was stimulated by the pre-anthesis HN treatment (Fig. 9a). In this condition, the vegetative biomass production was enhanced before anthesis (see Fig. S2B), triggering a high post-anthesis N demand from the plant on 1 mM NO_3_^−^ and thus explaining the stimulation of *BdNRT2A/B* expression. In the post-anthesis LN condition, the expression seemed also higher than in the control condition, despite being not statistically significant (Fig. 9a). In both situations, a high N demand from the shoots could explain this stimulation, presumably leading to a raise of the HATS activity. The increased demand is, however, likely due to two different physiological situations: an increased shoot biomass in the case of the pre-anthesis HN treatment (leading to an increased grain yield) and an N deficiency in the case of the post-anthesis LN treatment (leading to decreased grain yield and GNC). No effect was observed in the other conditions, which did not affect grain yield or GNC. This suggests a functional importance of the *BdNRT2A/B* in the responses to NO_3_^−^ availability. The expression of *BdNRT3*.*2* was insensitive to the treatments (Fig. 9b).

## Conclusion

Taken together, our data indicate that the developmental and physiological responses of Brachypodium (Bd21-3 accession) to N availability are comparable to previously studied C3 temperate cereals, such as wheat and barley. Specifically, NO_3_^−^ supply affects vegetative growth, shoot/root ratio, tiller formation, spike development, grain number per plant, grain yield and grain protein content. A high NHI, similar to what has been described in major cereals, was also observed. In addition to constitutive/inducible HATS and to a constitutive LATS, an inducible LATS was identified as a component of root NO_3_^−^ uptake systems, as previously highlighted in wheat.

Additionally, we revealed several specificities of Brachypodium Bd21-3 that are of special interest for crop improvement. For instance, understanding the basis of the unusually high GNC (despite a low N remobilization) in this model would be valuable towards an increased GPC in wheat. The post-anthesis NO_3_^−^ uptake seems to furnish a large part of grain N in Brachypodium Bd21-3, even under low supply condition. This will need to be further investigated under different experimental conditions and/or on several Brachypodium accessions. The knowledge gained from this could subsequently be used to improve NUE in cereals. Similarly, the identification of the molecular origin of the high constitutive HATS in Brachypodium Bd21-3 would be instrumental in enhancing the NO_3_^−^ uptake capacity of cereals in conditions of low availability. As a first insight, we identified *BdNRT2/3* genes potentially involved in constitutive and/or inducible HATS component.

Overall, the data identify the Bd21-3 accession of Brachypodium as a good model to decipher, at the physiological and molecular levels, the mechanisms involved in N metabolism in C3 temperate cereals. Based on our results, it will be beneficial to characterize a set of Brachypodium accessions, in order to distinguish traits that are specific to Bd21-3 from traits conserved in the entire species. Thus, our study provides the bases for candidate-gene and natural-variability approaches to identify molecular players affecting N nutrition and NUE in Brachypodium, towards the development of new cereal varieties with enhanced NUE in low N supply conditions.

## Materials and Methods

### Plant material and culture conditions

In this study, *Brachypodium distachyon* accession Bd21-3 was used. In the experiments, anthesis stage was defined as 7 days after emergence of the first spike^63^.

Plants grown on sand were cultivated in a growth chamber under the following conditions: 8/16 h light/dark cycle, 150 µmol photons.m^−2^.s^−1^ irradiation, 21/17 °C day/night temperature, 80% hygrometry. The basal nutrient medium contained 0.25 mM KH_2_PO_4_, 0.25 mM MgSO_4_, 27 µM Na-Fe(III)-EDTA, 243 µM (NH_4_)_6_Mo_7_O_24_, 0.4 µM H_3_BO_3_, 118 µM MnSO_4_, 10 µM de CuSO_4_ and 34.8 µM ZnSO_4_. For 0.1 mM NO_3_^−^ solution, the medium was supplemented with 0.1 mM KNO_3_, 0.35 mM K_2_SO_4_ and 0.25 mM CaCl_2_. For 2 mM NO_3_^−^ solution, the medium was supplemented with 1.75 mM KNO_3_, 0.125 mM Ca(NO_3_)_2_ and 0.125 mM CaCl_2_. For 10 mM NO_3_^−^ solution, the medium was supplemented with 5 mM KNO_3_, 2.5 mM Ca(NO_3_)_2_ and 0.2 mM NaCl. Plants were provided with nutrient solution 3 times a week in excess, and flow-through was discarded. Plants were harvested 35 days after sowing, 1 h after the start of the light period. Root system and individual tillers were weighed separately, frozen in liquid nitrogen and stored at −80 °C.

Plants grown in hydroponics were cultivated in a growth chamber under the following conditions: 18/6 h light/dark cycle, 250 µmol photons.m^−2^.s^−1^ irradiation, 22/19 °C day/night temperature, 65/90% hygrometry day/night. Grains were stratified 3–4 days at 4 °C in water, germinated in the dark at 20 °C in water for one week, and transferred to a hydroponic system composed of 15 L opaque tanks covered with perforated opaque lids. Each tank hosted 32 young plants at the beginning of the experiment, and down to 6 plants after anthesis to avoid a rapid depletion of the nutrient solution. Solution was fully renewed 2–3 times a week, and the steadiness of NO_3_^−^ concentration was validated using MQuant^TM^ Nitrate Test strips (Merck) and Nitrachek 404 quantification device (Quomed Ltd). Basal hydroponic solution was adapted from wheat studies^74,97^ and contained 1 mM KH_2_PO_4_, 2 mM MgSO_4_, 5 mM KCl, 50 µM Na-Fe(III)–EDTA, 4.5 µM MnCl_2_, 10 µM H_3_BO_3_, 0.7 µM ZnCl_2_, 0.4 µM CuSO_4_, 0.22 µM MoO_4_Na_2_. N-depleted medium was supplemented with 3.25 mM CaCl_2_. The 0.01 mM NO_3_^−^ medium was supplemented with 0.005 mM KNO_3_, 0.0025 mM Ca(NO_3_)_2_ and 3 mM CaCl_2_. The 0.1 mM NO_3_^−^ medium was supplemented with 0.05 mM Ca(NO_3_)_2_ and 3 mM CaCl_2_. The 0.2 mM NO_3_^−^ medium was supplemented with 0.1 mM KNO_3_, 0.05 mM Ca(NO_3_)_2_ and 3 mM CaCl_2_. The 1 mM NO_3_^−^ medium was supplemented with 0.5 mM Ca(NO_3_)_2_ and 3 mM CaCl_2_. The 2 mM NO_3_^−^ medium was supplemented with 1 mM KNO_3_, 0.5 mM Ca(NO_3_)_2_ and 3 mM CaCl_2_. The 10 mM NO_3_^−^ medium was supplemented with 5 mM KNO_3_, 2.5 mM Ca(NO_3_)_2_ and 2 mM CaCl_2_ and was deprived of KCl. For characterization on fresh material, plants were harvested at the middle of light period, roots were rinsed 1 min in 0.1 mM CaSO_4_, the plant parts were weighed separately, frozen in liquid nitrogen and stored at −80 °C until analyses. For end of cycle analyses, plants were harvested after full senescence, roots were rinsed 1 min in 0.1 mM CaSO_4_, roots, shoots and grains were collected separately and dried 48 h at 70 °C.

### Measurement of ^15^N, N and C contents and procedures for ^15^N labeling

Total N, C and ^15^N contents were quantified on 2–5 mg DW aliquots of ground tissues, dried at 70 °C for 48 h. The method is based on the Dumas combustion analysis which consist in a flash combustion of the samples in presence of He and O_2_ at 950 °C. The C and N elements are detected by gas chromatography on a FLASH 2000 Organic Elemental Analyzer (Thermo Fisher Scientific, Villebon, France). The ^15^N/^14^N isotopic ratio is subsequently quantified by a coupled mass spectroscope (Delta V advantage IRMS; Thermo Fisher Scientific, Villebon, France).

Protocol for NO_3_^−^ influx was adapted from Delhon and colleagues^98^. Plants grown in hydroponics were sequentially transferred to 0.1 mM CaSO_4_ for 1 min and to the basal nutrient solution (see above) supplemented with 3 mM CaCl_2_ and 0, 0.01, 0.025, 0.05, 0.1, 0.2, 0.5, 1, 2, 5 or 10 mM ^15^NO_3_^−^ (added as a mix of K^15^NO_3_^−^ and Ca(^15^NO_3_)_2_ at a molar ratio 2:1; 99% ^15^N atom) for 5 min. The roots were washed for 1 min in 0.1 mm CaSO_4_, shoots and roots were harvested separately, weighted and frozen in liquid nitrogen.

^15^N labeling procedure and calculation for the estimation of post-anthesis N fluxes were performed as previously described^71^. Five days ^15^N pulse was performed on 30 days-old plants (vegetative stage) by replacing the NO_3_^−^ source of 0.2 mM NO_3_^−^ medium by ^15^NO_3_^−^ (mix of K^15^NO_3_^−^ and Ca(^15^NO_3_)_2_ at a molar ratio 2:1; 2.5% ^15^N atom). Roots were subsequently rinsed 1 min in 0.1 mM CaSO_4_ and plants were transferred back to a non-labeled 0.2 mM NO_3_^−^ medium, before being transferred to 0.01, 0.2 or 10 mM NO_3_^−^ media at anthesis stage. Plants were harvested either at anthesis (to map the ^15^N distribution at the end of vegetative phase) or after full senescence of the plant. Roots, shoots and spikes were collected separately, dried 48 h at 70 °C and weighted.

### Measure of NO_3_^−^ content

Nitrate content was measured by a spectrophotometric method adapted from Miranda and colleagues^99^ after extraction in water of 10 mg FW or 1–2 mg DW ground tissues. The principle of this method is a reduction of nitrate by vanadium (III) through the acidic Griess reaction. The Griess reagent is a mixed solution of VCl_3_ (2.5% w/v), N-1-naphtylethylenediamine (0.05% w/v) and sulfanilamide (1% w/v) in 0.5 M HCl. It was added to plant extracts in equal proportion. After 2 hours of incubation at 60 °C, the diazoniium product was measured spectrophotometrically at 540 nm.

### Amino Acid and protein analyses

Free amino acids content and composition were determined after a three-step ethanol–water extraction (80%, 50% (v/v), then water at 4 °C) on 30 mg DW (for vegetative tissues) or 50 mg DW (for grains) of ground material, according to Ferrario-Méry and colleagues^100^. Free amino acid content was quantified by ninhydrin colorimetric analysis^101^. The amino acid composition was determined by ion-exchange chromatography using the AminoTac JLC-500/V amino acid analyzer according to the instructions of the manufacturer (JEOL (Europe), Croissy-sur-Seine, France).

Protein content was determined after extraction of 50 mg DW of grain powder in 0.062 M Tris-HCl pH 6.8, and 2% SDS^102^. Extractable proteins were quantified by the the RC DC™ (reducing agent and detergent compatible) protein assay kit (Bio-Rad Laboratories, Hercules, California, USA) based on the Lowry protocol. Samples of extractable proteins were separated on 10% SDS-PAGE gels and stained with Coomassie Brilliant Blue R250 (Sigma, St Louis, MO, USA).

### RNA extraction and qRT-PCR

Total RNAs were isolated using Trizol^®^ reagent (Ambion, Life Technologies), treated by DNase I (Thermo Scientific) and reverse-transcribed using oligo(dT)_18_ primer and RevertAid H Minus Reverse Transcriptase (Thermo Scientific), according to the manufacturer’s protocols. Reactions of qPCR were performed using LightCycler-FastStart DNA Master SYBR Green I kit (Roche) on a Realplex MasterCycler (Eppendorf), according to the manufacturer’s protocol. Primer sequences are specified in Supporting Information Table S4. Primers for housekeeping genes *BdEf1*α, *BdUBC18* and *BdUbi10* were selected from previous report^103^. Primer efficiency (eff) was determined at each run using a standard curve on a pool of cDNA. Normalized expression value was calculated using the formula: Normalized Relative Quantity = (1/eff^Ct^)_Hk_/(1/eff^Ct^)_GOI_, with Hk and GOI corresponding to Housekeeping gene and Gene Of Interest^104^, respectively. Statistical analyses of qRT-PCR data were performed by ANOVA on log2-transformed values^104^.

### Statistical analyses

All statistical tests have been performed with the R software, using the *multcomp*, *coin* and *RVAideMemoire* packages^105–107^. One-way ANOVA tests (qRT-PCR data) were used in conjunction with Tukey tests for the analysis of qRT-PCR data. Non-parametric one-way ANOVAs were performed using an approximate Fisher-Pitman permutation test, followed by a pairwise comparison test with the calculation of an adjusted *P*-value (fdr method).

## Supplementary information

Supplementary information

## Data Availability

All the relevant data supporting the findings are available from the corresponding author on reasonable request.
