# Supplementary material for: Developmental and physiological responses of Brachypodium distachyon to fluctuating nitrogen availability
Source: Sci Rep. 2019 Mar 7;9:3824. doi: 10.1038/s41598-019-40569-8 (PMC6405861; doi:10.1038/s41598-019-40569-8)
Supplement: Supplementary file 1 — Supplementary information [file 41598_2019_40569_MOESM1_ESM.pdf]

Developmental and physiological responses of *Brachypodium distachyon* to fluctuating nitrogen availability

David LC[+], Girin T[+]*, Fleurisson E, Phommabouth E, Mahfoudhi A, Citerne S., Berquin P, Daniel-Vedele F, Krapp A, Ferrario-Méry S.

Institut Jean-Pierre Bourgin, INRA, AgroParisTech, CNRS, Université Paris-Saclay, RD10, 78026 Versailles, France

[+] These authors contributed equally to this work

* corresponding author: thomas.girin@inra.fr

SUPPLEMENTARY INFORMATION

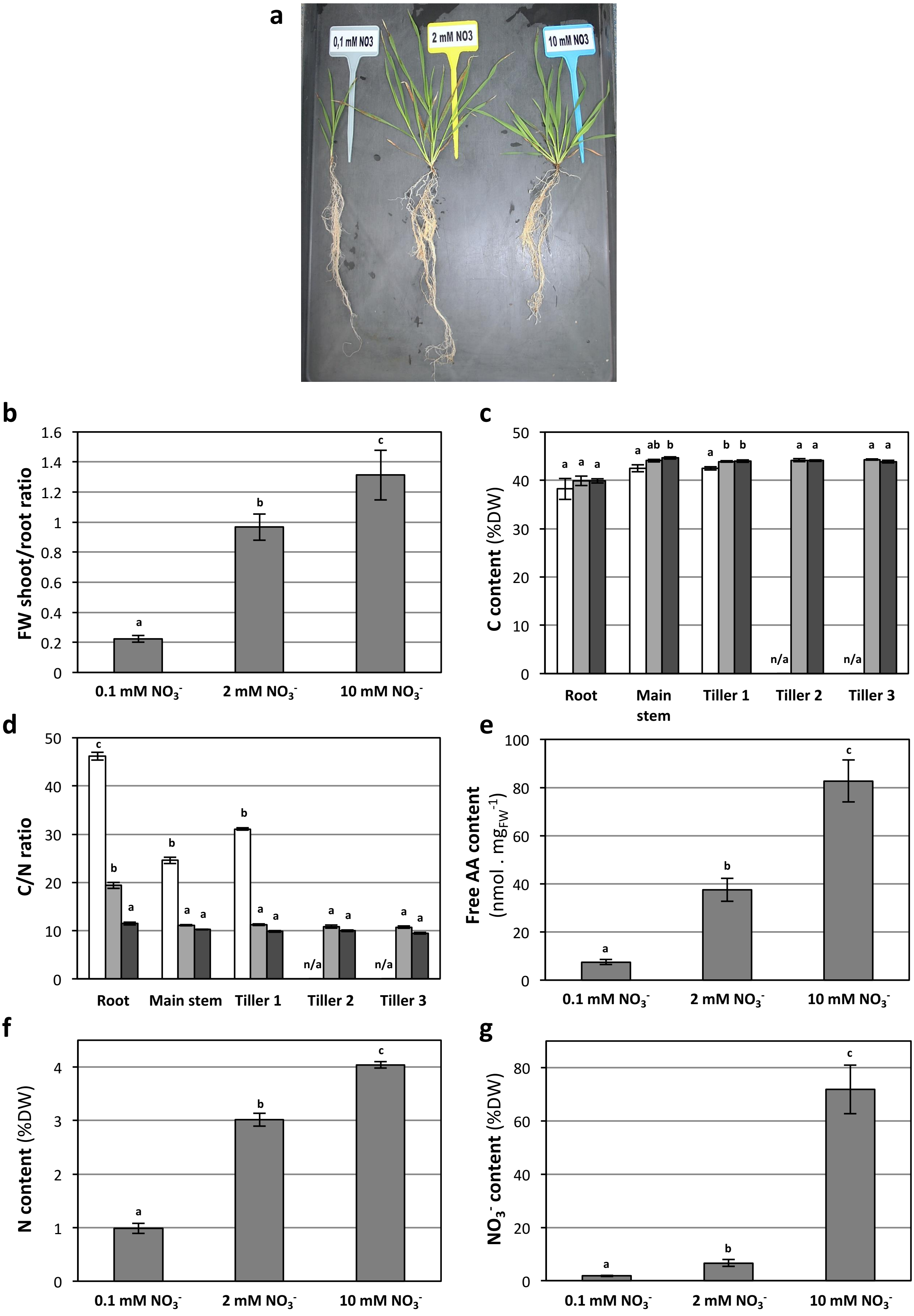

Figure S1: Effects of NO_3^- availability at vegetative stage. Plants were grown on sand for 35 days, watered with nutritive solution containing 0.1, 2 or 10 mM NO_3^-. **(a)** Pictures of representative plants grown on the 3 conditions. **(b)** Fresh weight shoot/root ratio in the 3 conditions. **(c)** C content and **(d)** C/N ratio of main stem and tillers. **(e)** Free AA content, **(f)** N content and **(g)** NO_3^- content of the whole plant in the 3 conditions. Values correspond to the mean of 4 or 5 biological replicates (2 plants each) +/-SD. Letters indicate statistical groups for each condition (panels b, e-g) or plant part (panels c,d) (Non-parametric ANOVA, $p < 0.05$). N/a: no data available, due to the lack of development of tillers 2 and 3 in the 0.1 mM NO_3^- condition.

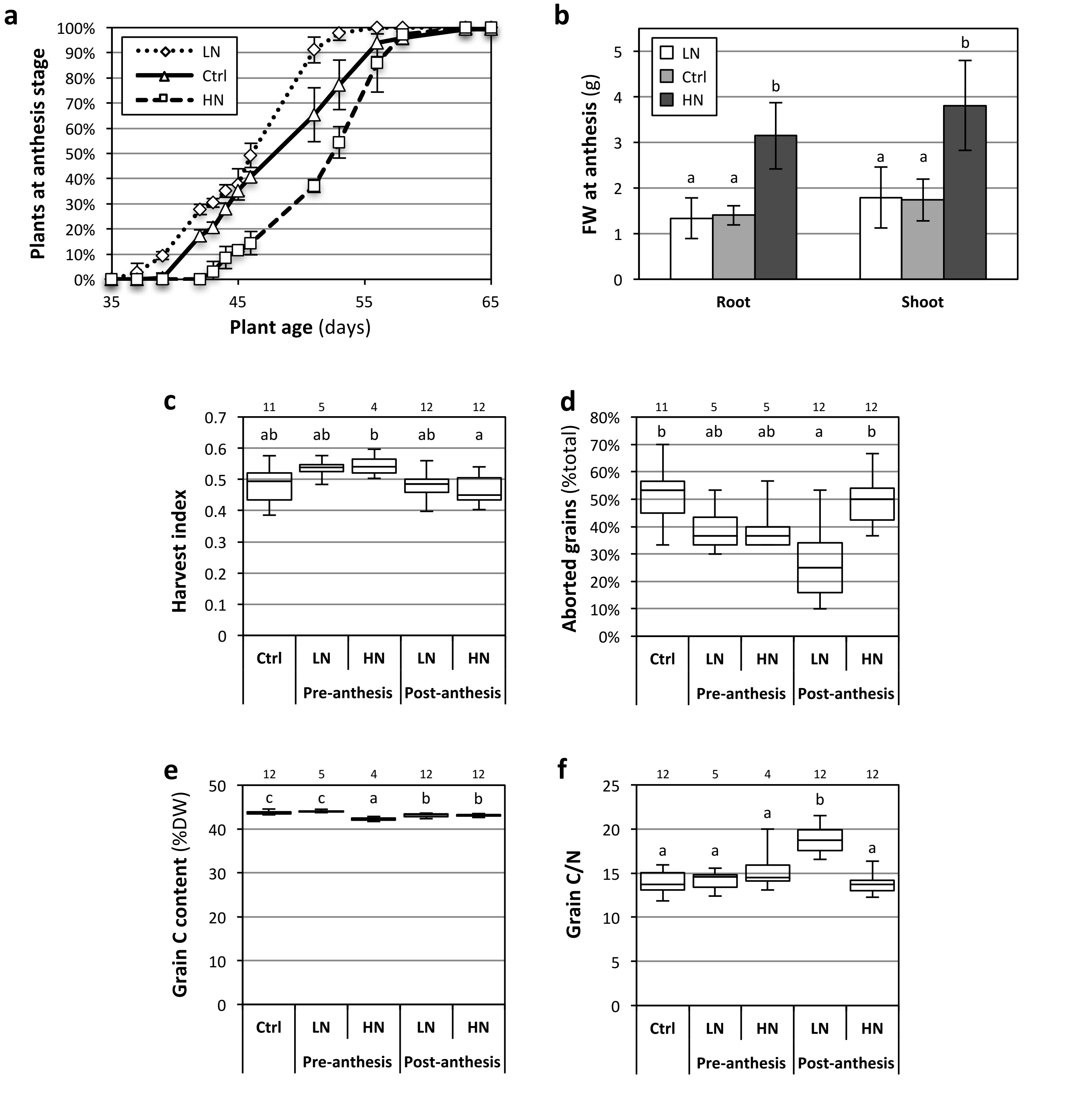

Figure S2: Effects of NO_3^- availability on anthesis characteristics and harvest index. Growing conditions and treatments were as described in Figure 2. LN, Ctrl and HN: 0.1, 1 and 10 mM NO_3^-, respectively. **(a)** Kinetics of anthesis. Values are means of 2 hydroponical tanks (LN condition; total of 43 plants), 8 tanks (C; 149 plants) and 2 tanks (HN; 35 plants) +/- SD. **(b)** Root and shoot FW at anthesis stage. Values are means of 15 plants +/-SD. **(c)** Grain harvest index (grain DW / total above-ground DW) at the end of the cycle. **(d)** Percentage of aborted grains. **(e)** Grain C content. **(f)** Grain C/N ratio. The boxplots represent minimum, 1st quartile, median, 3rd quartile and maximum values. Number of biological replicates for each condition are indicated above the boxplots. Letters indicate statistical groups (Non-parametric ANOVA, $p < 0.05$).

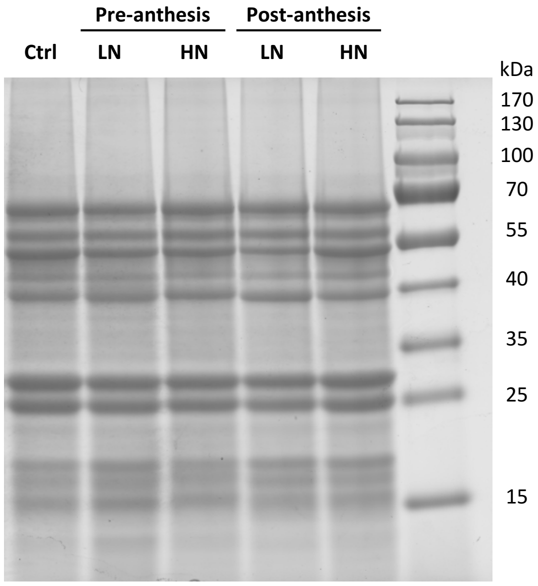

Figure S3: Effects of pre- and post-anthesis NO_3^- availability on grain protein composition analyzed by SDS PAGE electrophoresis. Growing conditions and treatments were as described in Figure 2. LN, Ctrl and HN: 0.1, 1 and 10 mM NO_3^-, respectively. Extractable proteins were separated on a 10% SDS PAGE gel and visualized by Coomassie blue staining. The same total protein quantity (50 μg) was loaded in each lane in order to compare the relative composition of proteins between the treatments. The estimated size of each molecular weight marker band (kDa) is indicated on the right of the figure.

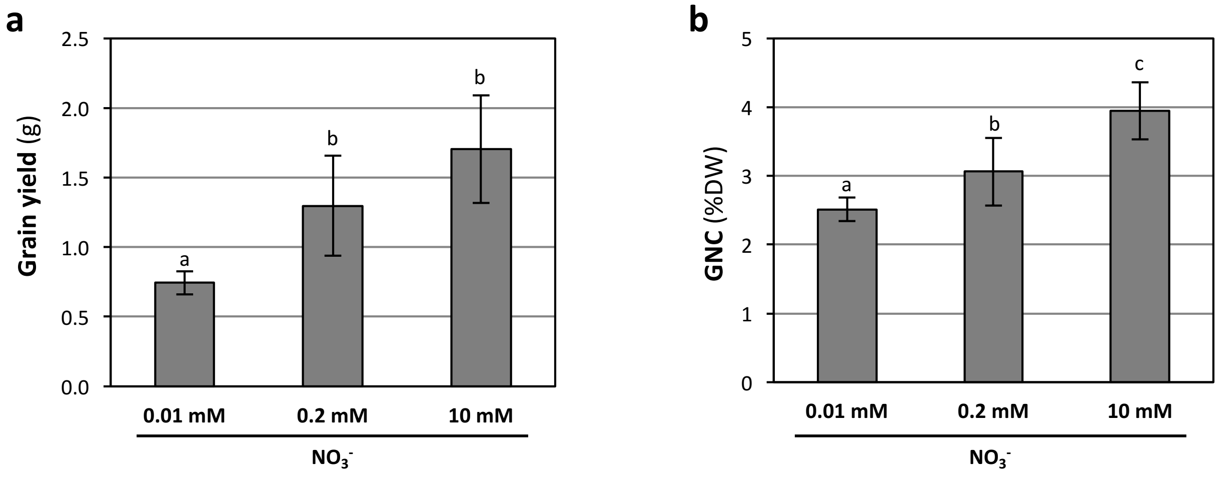

Figure S4: Effects of post-flowering NO_3^- availability on grain yield and Grain Nitrogen Content. Plants were grown in hydroponics on 0.2 mM NO_3^- and transferred at anthesis on 0.01, 0.2 or 10 mM NO_3^-. **(a)** Grain yield per plant. **(b)** Grain Nitrogen Content (GNC). Values correspond to the mean of 5 (0.01 mM NO_3^- condition) or 7 (0.2 and 10 mM NO_3^- conditions) biological replicates +/-SD. Letters indicate statistical groups (Non-parametric ANOVA, $p < 0.05$).

Table S1: Effects of pre- and post-anthesis NO_3^- availability on grain free AA composition

	Ctrl				LN pre-anthesis				HN pre-anthesis				LN post-anthesis				HN post-anthesis			
	Free AA content (nmol/mg$_{DW}$)		Free AA proportion (% total free AA)		Free AA content (nmol/mg$_{DW}$)		Free AA proportion (% total free AA)		Free AA content (nmol/mg$_{DW}$)		Free AA proportion (% total free AA)		Free AA content (nmol/mg$_{DW}$)		Free AA proportion (% total free AA)		Free AA content (nmol/mg$_{DW}$)		Free AA proportion (% total free AA)	
	mean value	*SD*	mean value	*SD*	mean value	*SD*	mean value	*SD*	mean value	*SD*	mean value	*SD*	mean value	*SD*	mean value	*SD*	mean value	*SD*	mean value	*SD*
**ALA**	1.33	*0.20*	5.41%	*0.73%*	1.37	*0.27*	6.55%	*1.39%*	1.70	*0.41*	6.68%	*1.12%*	2.47	*0.84*	10.27%	*3.00%*	1.02	*0.17*	6.67%	*1.18%*
**ARG**	1.58	*0.71*	6.13%	*1.45%*	1.27	*0.66*	5.60%	*1.33%*	1.15	*0.35*	4.55%	*1.19%*	0.70	*0.21*	2.91%	*0.71%*	1.04	*0.44*	6.44%	*1.66%*
**ASN**	10.76	*2.14*	43.49%	*3.18%*	8.73	*3.95*	39.25%	*7.21%*	8.37	*2.37*	33.25%	*8.59%*	6.40	*1.52*	26.79%	*5.45%*	6.79	*1.03*	43.80%	*3.12%*
**ASP**	1.45	*0.20*	5.94%	*0.75%*	1.56	*0.51*	7.23%	*1.02%*	1.62	*0.26*	6.39%	*0.46%*	1.11	*0.20*	4.68%	*0.87%*	0.91	*0.35*	5.73%	*1.77%*
**GABA**	0.34	*0.08*	1.39%	*0.31%*	0.35	*0.16*	1.55%	*0.33%*	0.54	*0.35*	2.09%	*1.19%*	0.36	*0.07*	1.49%	*0.23%*	0.26	*0.03*	1.68%	*0.26%*
**GLN**	1.50	*0.63*	5.83%	*1.25%*	1.26	*0.69*	5.46%	*1.38%*	1.87	*0.42*	7.35%	*1.06%*	2.50	*0.36*	10.49%	*1.10%*	0.77	*0.37*	4.70%	*1.48%*
**GLU**	1.19	*0.25*	4.78%	*0.28%*	1.04	*0.26*	4.85%	*0.58%*	1.34	*0.43*	5.37%	*2.02%*	0.76	*0.13*	3.16%	*0.43%*	0.90	*0.13*	5.83%	*0.54%*
**GLY**	0.32	*0.16*	1.28%	*0.48%*	0.24	*0.03*	1.16%	*0.25%*	0.25	*0.02*	0.98%	*0.09%*	0.32	*0.10*	1.36%	*0.42%*	0.20	*0.03*	1.30%	*0.25%*
**ILE**	0.28	*0.07*	1.13%	*0.19%*	0.26	*0.06*	1.24%	*0.29%*	0.34	*0.15*	1.34%	*0.50%*	0.42	*0.07*	1.79%	*0.32%*	0.18	*0.03*	1.15%	*0.20%*
**LEU**	0.29	*0.07*	1.19%	*0.13%*	0.27	*0.05*	1.27%	*0.27%*	0.37	*0.17*	1.43%	*0.59%*	0.39	*0.06*	1.62%	*0.26%*	0.18	*0.04*	1.19%	*0.19%*
**LYS**	0.32	*0.11*	1.25%	*0.17%*	0.28	*0.13*	1.23%	*0.21%*	0.30	*0.08*	1.18%	*0.14%*	0.42	*0.03*	1.76%	*0.08%*	0.19	*0.07*	1.18%	*0.29%*
**ORN**	0.15	*0.14*	0.55%	*0.45%*	0.06	*0.04*	0.25%	*0.08%*	0.07	*0.03*	0.21%	*0.14%*	0.12	*0.04*	0.50%	*0.12%*	0.04	*0.02*	0.24%	*0.07%*
**PHE**	0.28	*0.08*	1.11%	*0.13%*	0.27	*0.07*	1.26%	*0.34%*	0.33	*0.09*	1.28%	*0.24%*	0.25	*0.05*	1.05%	*0.23%*	0.16	*0.03*	1.02%	*0.13%*
**PRO**	2.95	*0.77*	11.81%	*1.68%*	3.17	*1.19*	14.70%	*4.30%*	4.38	*1.84*	16.87%	*5.34%*	0.95	*0.30*	3.92%	*1.01%*	1.81	0.63	11.38%	*2.41%*
**SER**	0.79	*0.36*	3.10%	*0.98%*	0.56	*0.18*	2.54%	*0.22%*	0.80	*0.26*	3.11%	*0.74%*	1.56	*0.19*	6.56%	*0.96%*	0.41	*0.03*	2.67%	*0.34%*
**THR**	0.49	*0.08*	2.00%	*0.23%*	0.43	*0.14*	1.99%	*0.26%*	0.58	*0.19*	2.24%	*0.54%*	0.78	*0.08*	3.28%	*0.44%*	0.31	*0.04*	1.99%	*0.33%*
**TRP**	0.07	*0.03*	0.19%	*0.19%*	0.05	*0.04*	0.24%	*0.17%*	0.28	*0.49*	1.17%	*2.10%*	3.21	*0.42*	13.50%	*1.88%*	0.03	*0.00*	0.17%	*0.05%*
**TYR**	0.15	*0.05*	0.60%	*0.21%*	0.14	*0.03*	0.66%	*0.12%*	0.17	*0.06*	0.69%	*0.19%*	0.20	*0.04*	0.85%	*0.17%*	0.10	*0.01*	0.64%	*0.17%*
**VAL**	0.70	*0.15*	2.83%	*0.17%*	0.65	*0.23*	2.97%	*0.68%*	0.98	*0.54*	3.81%	*1.78%*	0.95	*0.09*	4.02%	*0.57%*	0.36	*0.20*	2.21%	*1.24%*

Table S2: raw data for N fluxes calculation (Figure 5)

Plant number	Harvesting stage	NO_3^- condition	Note	Root DW (mg)	Root 15N abundance (%N)	Root N content (%DW)	Shoot DW (mg)	Shoot 15N abundance (%N)	Shoot N content (%DW)	Spike DW (mg)	Spike 15N abundance (%N)	Spike N content (%DW)
1	Reproductive	0.01 mM		71.7	0.382	1.22	94.6	0.367	0.82	148.0	0.366	1.56
2	Reproductive	0.01 mM	For natural ^{15}N	141.7	0.368	1.84	345.7	0.368	1.05	374.3	0.367	1.79
3	Reproductive	0.2 mM	abundance	247.2	0.367	3.08	831.6	0.368	1.96	720.5	0.366	2.18
4	Reproductive	0.2 mM	determination.	113.5	0.368	3.28	274.7	0.367	1.99	336.0	0.366	2.30
5	Reproductive	10 mM	No ^{15}N labeling.	207.3	0.366	4.10	543.9	0.365	2.73	457.7	0.364	2.43
6	Reproductive	10 mM		154.2	0.366	4.13	500.6	0.366	2.40	426.5	0.365	2.49
7	Anthesis	0.2 mM		93.4	0.574	2.72	380.4	0.692	1.37	113.1	0.685	2.09
8	Anthesis	0.2 mM	For ^{15}N	145.9	0.906	2.24	520.3	1.135	1.84	73.0	1.006	2.40
9	Anthesis	0.2 mM	distribution at	84.2	0.813	2.30	292.2	1.094	1.61	86.2	0.965	2.22
10	Anthesis	0.2 mM	anthesis.	91.8	0.856	2.73	358.2	1.076	2.07	117.9	0.978	2.32
11	Anthesis	0.2 mM		77.5	0.874	2.70	290.0	1.242	2.20	117.6	1.076	2.45
12	Reproductive	0.01 mM		184.1	0.538	1.59	603.1	0.569	0.79	789.6	0.641	2.32
13	Reproductive	0.01 mM	For ^{15}N	185.4	0.557	2.11	633.4	0.636	0.84	750.8	0.798	2.43
14	Reproductive	0.01 mM	distribution at	91.9	0.653	2.27	476.9	0.791	0.67	765.3	0.804	2.71
15	Reproductive	0.01 mM	end of cycle.	101.3	0.545	1.54	388.3	0.628	0.84	601.8	0.626	2.43
16	Reproductive	0.01 mM		197.9	0.535	1.80	618.9	0.626	0.67	812.0	0.623	2.68
17	Reproductive	0.2 mM		225.0	0.404	3.49	1142.0	0.417	2.45	1384.0	0.510	3.76
18	Reproductive	0.2 mM		185.0	0.443	2.87	906.0	0.503	1.63	1491.0	0.469	2.79
19	Reproductive	0.2 mM	For ^{15}N	105.0	0.490	2.14	684.0	0.543	0.80	954.0	0.542	2.34
20	Reproductive	0.2 mM	distribution at	159.0	0.488	2.06	748.0	0.496	1.14	1323.0	0.493	2.88
21	Reproductive	0.2 mM	end of cycle.	303.0	0.415	2.72	1293.0	0.432	1.82	1810.0	0.439	3.60
22	Reproductive	0.2 mM		186.0	0.415	3.24	669.0	0.450	1.61	829.0	0.546	3.18
23	Reproductive	0.2 mM		69.0	0.515	1.36	276.0	0.580	0.91	532.0	0.485	2.89
24	Reproductive	10 mM		217.0	0.409	4.00	845.0	0.403	3.52	1003.0	0.401	4.09
25	Reproductive	10 mM		229.0	0.421	3.78	1245.0	0.425	2.42	1858.0	0.425	4.27
26	Reproductive	10 mM	For ^{15}N	177.0	0.436	2.75	1131.0	0.419	2.78	1631.0	0.427	3.91
27	Reproductive	10 mM	distribution at	118.0	0.456	3.50	956.0	0.450	2.44	1404.0	0.441	3.06
28	Reproductive	10 mM	end of cycle.	90.0	0.475	1.73	575.0	0.433	2.51	1166.0	0.432	3.97
29	Reproductive	10 mM		231.0	0.420	2.98	1327.0	0.403	2.64	2241.0	0.391	4.05
30	Reproductive	10 mM		176.0	0.404	4.30	1068.0	0.407	2.76	1928.0	0.401	4.29

Table S3: statistical tests of N fluxes (Figure 5)

Experimental design and results are presented in Figure 5.
Letters indicate statistical groups (Kruskal-Wallis H test, $p < 0.05$).
Comparisons are presented per line (ie: same letter on different lines do not mean same group).

Table S3(A) : comparison between N fluxes intensities for each treatment

Treatment	Remobilization from roots	Remobilization from shoots	Remobilization to spikes	Influx to roots	Influx to shoots	Influx to spikes
0.01 mM NO_3^-	c	d	b	ac	a	b
0.2 mM NO_3^-	b	c	d	bcd	acd	a
10 mM NO_3^-	c	d	e	de	a	b

Table S3(B) : comparison of end of cycle N quantities between plant parts for each treatment

Treatment	Roots	Shoots	Spikes
0.01 mM NO_3^-	a	a	b
0.2 mM NO_3^-	a	a	b
10 mM NO_3^-	a	b	c

Table S3(C) : comparison of N fluxes and quantities between treatments

		0.01 mM NO_3^-	0.2 mM NO_3^-	10 mM NO_3^-
Total fluxes at the plant level	Remob	b	a	a
	Uptake	a	b	c
Remobilization	from roots	a	a	a
	from shoots	c	b	a
	to spikes	b	b	a
Influx	to roots	a	a	a
	to shoots	a	a	b
	to spikes	a	b	c
End of cycle N quantities	Roots	a	a	a
	Shoots	a	a	b
	Spikes	a	b	c
End of cycle N distribution (% of total plant N)	Roots	a	a	a
	Shoots	a	ab	b
	Spikes	a	a	a

Table S4: primers used for qRT-PCR analyses

Name symbol	Gene number	Primer	Sequence (5'->3')
BdNRT2A/B	*Bradi3g01270.1*	Forward	GTCGGGTTCCATCTTCTCG
	Bradi3g01250.1	Reverse	CCGATGATCTTGCTGTTGAA
BdNRT2C	*Bradi3g01290.1*	Forward	AAGTACCCCACAAGCAAAGG
		Reverse	TCCCTTGCTCTTCTCCTCA
BdNRT2D	*Bradi3g01277.1*	Forward	GCTTCGGCTTGGCTAATATC
		Reverse	ACCATGGATACGATGGAGGT
BdNRT3.1	*Bradi3g47710.1*	Forward	TACGGCCAGACCAAGCTC
		Reverse	CCTTGGCAAGTTGCATTATC
BdNRT3.2	*Bradi3g47720.1*	Forward	TCGCCTTCTTCTTCTTCATC
		Reverse	GCCTTGCAATAGATTGCTGT
Ubi10	*DV484269*	Forward	TCCACACTCCACTTGGTGCT
		Reverse	GAGGGTGGACTCCTTTTGGA
EF1α	*DV482887*	Forward	CCATCGATATTGCCTTGTGG
		Reverse	GTCTGGCCATCCTTGGAGAT
UBC18	*DV481689*	Forward	GGAGGCACCTCAGGTCATTT
		Reverse	ATAGCGGTCATTGTCTTGCG
